# Mutual proximity graphs for improved reachability in music recommendation

**DOI:** 10.1080/09298215.2017.1354891

**Published:** 2017-08-03

**Authors:** Arthur Flexer, Jeff Stevens

**Affiliations:** ^a^ Austrian Research Institute for Artificial Intelligence (OFAI), Austria.; ^b^ George Mason University, Virginia, USA.

**Keywords:** Music recommendation, graphs, hubness, curse of dimensionality

## Abstract

This paper is concerned with the impact of hubness, a general problem of machine learning in high-dimensional spaces, on a real-world music recommendation system based on visualisation of a k-nearest neighbour (knn) graph. Due to a problem of measuring distances in high dimensions, hub objects are recommended over and over again while anti-hubs are nonexistent in recommendation lists, resulting in poor reachability of the music catalogue. We present mutual proximity graphs, which are an alternative to knn and mutual knn graphs, and are able to avoid hub vertices having abnormally high connectivity. We show that mutual proximity graphs yield much better graph connectivity resulting in improved reachability compared to knn graphs, mutual knn graphs and mutual knn graphs enhanced with minimum spanning trees, while simultaneously reducing the negative effects of hubness.

## 1. Introduction

In graph theory, Ozaki, Shimbo, Komachi, and Matsumoto (2011) have observed that knn graphs^1^ often produce hubs, i.e. vertices with extremely high numbers of edges to other vertices. The same authors also made the connection to the phenomenon of ‘hubness’, which is a term used to describe a general problem of learning in high-dimensional spaces (Radovanović, Nanopoulos, & Ivanović, 2010). Hubness has been first noted in music information retrieval (MIR) (Aucouturier & Pachet, 2004), but is now acknowledged as a general machine learning problem and a new aspect of the curse of dimensionality (Radovanović et al., 2010; Schnitzer, Flexer, Schedl, & Widmer, 2012). While hubs appear very close to many other vertices, anti-hubs present as distant to all vertices. Both phenomena arise from the concentration of distance measures (Francois, Wertz, & Verleysen, 2007) in high-dimensional spaces.

This hubness phenomenon has been shown to negatively impact a real-world music recommendation system which has been built by our research team. This system uses visualisation of a knn graph^2^ to recommend music via a web interface. In previous work (Gasser & Flexer, 2009; Flexer, Gasser, & Schnitzer, 2010) we were able to show that hubness causes some songs to never appear in any nearest neighbour list, because hubs crowd the nearest neighbour’s lists and are therefore repeatedly recommended. As a result, only about two-thirds of the songs are reachable in the recommendation interface, i.e. over a third of the songs are never recommended. Further analysis of the knn graph shows that only less than a third of the songs are likely to be recommended, since only those are part of one large strongly connected subgraph. We have already applied ‘mutual proximity’ (Schnitzer et al., 2012), a hubness reduction method, to improve this situation, but have not yet explored this topic in graph theoretical terms.

This paper presents a thorough analysis of the properties of this music recommendation system based on knn graphs, as well as three alternative graph construction methods, all in the light of hubness. We present related work in Section 2, the data for the empirical evaluation in Section 3, the graph construction and evaluation methods in Section 4, the results of our analysis in Section 5 and finally discuss and conclude in Sections 6 and 7. This paper is an extended version of a previous workshop contribution (Flexer & Stevens, 2016), with the main novel contributions being introduction of an additionalgraph construction method using a minimum spanning tree, a random walk analysis and overall increased depth of treatment.

## 2. Related work

In our overview of related work, we concentrate on the hubness problem, with an emphasis on its relation to audio-based music retrieval and graph analysis. The music recommendation literature is rich in comprehensive resources such as Oscar Celma’s seminal book (Celma, 2010), surveys on recommendation via collaborative filtering and content-based models (Song, Dixon, & Pearce, 2012), via context-based information (Knees & Schedl, 2013), as well as a treatment of the topic in a recent road map on music information research (Serra et al., 2013). We refer the reader to the above resources for excellent perspectives on music recommendation technology. Note that our work deals with content-based music recommendation computed from audio information directly, where the recommendation approach is based on graphical analysis of interactively built playlists of songs (Section 3).

Before discussing the hubness phenomenon, we would like to make clear that the notion of hub vertices in networks (Barabási & Albert, 1999) predates the notion of hubness in MIR (Aucouturier & Pachet, 2004) and machine learning (Radovanović et al., 2010). Given the focus of this paper, we will start our review of related literature with MIR and machine learning, and then proceed to the graph literature.

In MIR, hubness was first noted as a problem in audio-based music recommendation (Aucouturier & Pachet, 2004), more specifically that certain songs were being recommended conspicuously often in nearest neighbour-based playlists. Hubness has since gained attention in the machine learning community, where it has been described as a new aspect of the curse of dimensionality and a general problem of learning in high-dimensional spaces (Radovanović et al., 2010; Schnitzer et al., 2012). Hubness is related to the phenomenon of concentration of distances, where all pairwise distances are approximately the same for dimensionality approaching infinity (Francois et al., 2007). Radovanović et al. (2010) presented the argument that for any finite dimensionality, some points are expected to be closer to the centre of all data than other points, and are at the same time closer, on average, to all other points. Such points closer to the centre have a high probability of being hubs, i.e. of appearing in nearest neighbour lists of many other points. Points which are farther away from the centre have a high probability of being anti-hubs, i.e. points that never appear in any nearest neighbour list. It should be noted that the same authors, in their analysis of the influence of hubness on nearest neighbour methods, already made the connection to *k*-nearest neighbour graphs.

Along the same lines, the relationship between hubness and graph theory has also been established by viewing a recommender system as a directed graph (Celma, 2010; Seyerlehner, Flexer, & Widmer, 2009). Every item in a database is a vertex of the graph and every recommendation of an item $v_{j}$ from an item $v_{i}$ is represented as a directed edge $e_{ij}$ leading from $v_{i}$ to $v_{j}$ thereby constructing a nearest neighbour graph (Eppstein, Paterson, & Yao, 1997). This point of view connects the hubness problem to the rich literature on graph theory and complex network analysis (see Cohen & Havlin, 2010 for an overview). It also links to results in MIR that demonstrate hubs are distributed along a scale-free distribution (Aucouturier & Pachet, 2008), which is an important property of many real-world graphs. So-called ‘scale-free networks’ contain hub vertices which have many more connections than other vertices and therefore the network as a whole exhibits a power-law distribution in the number of links incident to a node (Barabási & Albert, 1999). In such networks, hubs guarantee a short path length between all vertices and as such are seen beneficial rather than harmful because they promote network resiliency. A graph theory-based analysis of an existing music recommendation service has shown limited accessibility of its audio catalogue due to hub and anti-hub nodes (Flexer et al., 2010), where anti-hub vertices are the logical opposites of hubs, i.e. vertices with in-degrees of zero. Related studies (Celma, 2010; Celma & Cano, 2008) on artist similarity showed that artist similarity graphs based on audio content, collaborative filtering or expert advice all exhibit the hubness characteristic of scale-free networks.

Hubness has also been shown to have a negative impact on many more tasks including classification (Dinu, Lazaridou, & Baroni, 2015; Radovanović et al., 2010; Shigeto, Suzuki, Hara, Shimbo, & Matsumoto, 2015), regression (Buza, Nanopoulos, & Nagy, 2015), nearest neighbour-based recommendation (Flexer, Schnitzer, & Schlüter, 2012) and retrieval (Schnitzer, Flexer, & Tomašev, 2014), clustering (Schnitzer & Flexer, 2015; Tomašev, Radovanović, Mladenić, & Ivanović, 2013), visualisation (Flexer, 2015) and outlier detection (Flexer, 2016; Radovanović, Nanopoulos, & Ivanović, 2015). Hubness also affects data from diverse domains including multimedia (text, music, images, speech), biology and general machine learning (see Feldbauer & Flexer, 2016; Radovanović et al., 2010; Schnitzer et al., 2012 for large-scale empirical studies). It is important to note that the degree of distance concentration and hubness is linked to the intrinsic rather than extrinsic dimension of the data space. Whereas the extrinsic dimension is the actual number of dimensions of a data space, the intrinsic dimension is the, often much smaller, number of degrees of freedom of the submanifold in which the data space can be represented (Francois et al., 2007).

Basically three different approaches have been proposed to reduce hubness and its negative effects: re-scaling (Schnitzer et al., 2012; Tomašev & Mladenić, 2014) of the distance space, centreing of the data (Hara et al., 2015), using $l^{p}$ norms different than Euclidean $l^{2}$ norm (Flexer & Schnitzer, 2015). Of special interest for this paper are re-scaling approaches like mutual proximity (MP) and local scaling (LS), which aim at repairing asymmetric non-mutual nearest neighbour relations. The asymmetric relations are a direct consequence of the presence of hubs. A hub *y* is the nearest neighbour of *x*, but the nearest neighbour of the hub *y* is another point *a* (a≠x). This is because hubs are by definition nearest neighbours to very many data points but only one data point can be the nearest neighbour to a hub. The principle of the scaling algorithms is to rescale distances to enhance symmetry of nearest neighbours. A small distance between two objects should be returned only if their nearest neighbours are mutual. Application of MP and LS resulted in a decrease of hubness and an accuracy increase in *k*-nearest neighbour classification on 30 real-world data-sets including text, image and music data (Schnitzer et al., 2012).

Yet another approach towards hubness reduction, and the most relevant work for this paper, is an application of mutual *k*-nearest neighbour graphs to semi-supervised classification of natural language data (Ozaki et al., 2011). The authors observe that knn graphs often produce hubs and that mutual knn graphs can reduce this hub effect and at the same time increase classification accuracy. In what follows, we will essentially apply the mutual knn graphs proposed by the authors in a music recommendation setting and compare it to mutual proximity graphs, which we will introduce in Section 4.1. This connects to our own previous work on using mutual proximity for reducing hubness in music recommendation (Schnitzer et al., 2012) by adding a graph theoretic perspective that has been missing so far.

## 3. Data

For our analysis, we use data from the real-world music discovery system FM4 Soundpark (http://fm4.orf.at/soundpark), a web platform run by the Austrian public radio station FM4, where artists can upload and present their music free of charge. Website visitors can listen to and download all the music at no cost, with most recent uploads being displayed at the top of the website. Whereas chronological publishing is suitable to promote new releases, older releases tend to quickly disappear from the user’s attention. In the case of the FM4 Soundpark, this has resulted in users mostly listening to music that is advertised on the front-page, and therefore missing the full breadth of the music database. To allow a more intuitive and appealing access to the full database regardless of the song’s publication date, we implemented a recommendation system using a content-based music similarity measure (Gasser & Flexer, 2009). This similarity measure is based on timbre information computed from the audio.

Each time a song is uploaded,^3^ 2 min of raw 22,050 Hz mono audio signals from the centre of the submission is used for analysis and similarity ranking. We divide the raw audio data into overlapping frames of short duration and transform them to Mel Frequency Cepstrum Coefficients (MFCC), resulting in a spectrally smoothed and perceptually meaningful representation of the audio signal. MFCCs are a standard technique for computation of spectral similarity in music analysis (see e.g. Logan, 2000). The frame size for computation of MFCCs for our experiments was 46.4 ms (1024 samples). We used the first 20 MFCCs for all our experiments. The MFCCs of each song are modelled via a single Gaussian with full covariance matrix (Mandel & Ellis, 2005). To compute a distance value between two Gaussians, the symmetrised Kullback–Leibler (SKL) divergence is used (see Gasser & Flexer, 2009 for more details on both MFCCs and SKL). This results in an N×N symmetric distance matrix *D* for the data-set, with N=7665 songs. The data-set of 7665 songs is a snapshot of the constantly growing Soundpark database. As new songs continue to be added, new distance measures are computed and the knn graph is updated (some edges added, and some deleted).

As previously mentioned, 20 MFCCs are used to generate a Gaussian song model. Each of these models contains 190 covariances and 20 mean values, resulting in an extrinsic dimension of 210 dimensions. The reader is referred to Mandel and Ellis (2005) for specifics. The intrinsic dimensionality (Levina & Bickel, 2005) of the data computed from the distance matrix *D* is 14. Whereas this may sound small, previous work (Schnitzer et al., 2012) has shown that data with intrinsic dimensionality as low as 9 can already be negatively impacted by hubness.

The database of songs is organised in a rather coarse genre taxonomy, where the artists themselves are able to choose which of six genre labels best describe their music. The artists are allowed to choose one or two of the genre labels, hence the following percentages add up to more than 100%: 37.6% of all songs belong to genre Pop, 46.3% to Rock, 44.0% to Electronica, 14.3% to Hip-Hop, 19.7% to Funk, 5.3% to Reggae (see Flexer et al., 2010 for more detail concerning the database).

**Figure 1.**
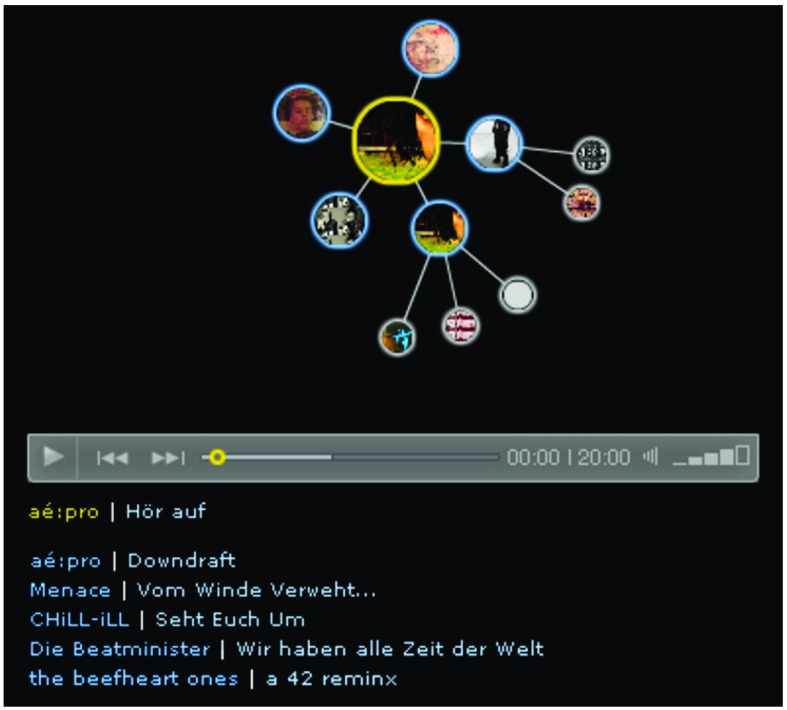
Soundpark web player showing recommendations as a visualisation of the underlying knn graph and as a text list.

The recommendation system has been implemented as a web player which can be accessed from within an artist’s web page on the Soundpark website by clicking on one of the artist’s tracks. In addition to offering the usual player interface (start, stop, skipping forward/backward), the system recommends songs similar to the selected track in a text list and in a graph-based visualisation (see Figure 1). The graph visualisation displays an interactively constructed nearest neighbour graph (number of nearest neighbours = 5), where a song is shown as a large centred circle with edges connecting it to the most similar (nearest neighbor) songs. Circle sizes for similar songs are inversely proportional to edge distance from the selected song, to a maximum two. For every song a user clicks, its nearest neighbours are displayed, thereby interactively constructing a visualisation of the knn graph, with circles representing vertices and lines representing edges. Vertices having an edge distance greater than two from the central starting vertex are trimmed. Songs which are nearest neighbours to more than one other song in the display are only shown the first time they appear as a nearest neighbour, thereby making it possible that less than five neighbours are displayed for some of the songs. The original Soundpark system was characterised by users listening mainly to the latest uploaded songs. However, introduction of this audio-based recommender system resulted in an increase of distinct song accesses more evenly distributed across the entire music catalogue (see Gasser & Flexer, 2009 for an analysis of download statistics and user behaviour).

## 4. Methods

The user interface of the music recommender has been implemented as a visualisation of a knn graph showing the k=5 most similar songs to the currently selected track. In what follows we will describe the construction of the knn graph and three alternative construction methods in Section 4.1. Measures to evaluate these graphs are presented in Section 4.2.

### 4.1. Graph construction

A graph G=(V,E) is defined via a finite set of vertices $V=\{v_{1},v_{2},\cdots ,v_{n}\}$ and edges $E=\{e_{1},e_{2},\cdots ,e_{m}\}$. In our case, the vertices correspond to songs displayed in the music recommender interface and the edges connect similar songs.

*k*-**nearest neighbour graphs (knn):** knn graphs are a very common graph construction technique, where an edge $e_{ij}$ is placed between $v_{i}$ and $v_{j}$ if $v_{j}$ is among the *k* nearest neighbours of $v_{i}$. We use the distance matrix *D* defined in Section 3 to compute an adjacency matrix *A*. If, according to *D*, a song with the index *j* is among the five nearest neighbours of a song with index *i*, then $A_{ij}=1$, otherwise $A_{ij}=0$. An edge $e_{ij}$ exists between two vertices $v_{i}$ and $v_{j}$ if $A_{ij}=1$. Please note that the adjacency matrix *A* is not symmetric and the resulting graph is therefore a directed knn graph. For a knn graph, the out-degree $d^{+}\left(v_{i}\right)$ of a vertex $v_{i}$, i.e. the number of edges having origin $v_{i}$, is always equal to *k* (k=5 in this work). The in-degree $d^{-}\left(v_{i}\right)$, i.e. the number of edges with destination $v_{i}$, is in the interval [0,N-1], with *N* being the number of vertices.

**Mutual** *k*-**nearest neighbour graphs (muknn):** muknn graphs (see e.g. Ozaki et al., 2011) are a simple extension of knn graphs wherein only bidirectional edges are retained, i.e. an edge $e_{ij}$ exists only if $A_{ij}=A_{ji}=1$, which indicates song *j* and song *i* are in each other’s nearest neighbour lists. The resulting muknn graph is therefore a subset of the corresponding knn graph and versions of it have already been applied to reduce hubness (Ozaki et al., 2011). Both in- and out-degrees of all vertices are in the interval [0, *k*], with the additional constraint that $d^{+}\left(v_{i}\right)=d^{-}\left(v_{i}\right)$ for all vertices in the muknn graph.

**Mutual** *k*-**nearest neighbour graphs plus minimum spanning tree (muknn+msp):** many edges can be deleted during construction of muknn graphs, sometimes yielding poorly connected vertices. It has already been suggested (Ozaki et al., 2011) to add a minimum spanning tree (Kruskal, 1956) (msp) to the muknn graph to improve connectivity. A msp is a graph of minimum weight connecting all vertices. It is a subset of an edge-weighted complete graph without any self-loops or cycles. We build a msp using the distance matrix *D* as input, i.e. the edge weights correspond to the distances between songs as described in Section 3. We use the classical Prim’s algorithm (Prim, 1957) to construct the msp. The resulting msp is an undirected graph, or equivalently a bidirectional graph with $A_{ij}=A_{ji}=1$, i.e. undirected edges are replaced by two corresponding directed edges. The compound muknn+msp graph consists of all edges which are either part of the muknn or msp graph. Both in- and out-degrees of all vertices are in the interval [1,N-1], with the lower limit of one being due to the fact that now every vertex is part of one fully connected graph. The additional constraint that $d^{+}\left(v_{i}\right)=d^{-}\left(v_{i}\right)$ for all vertices still holds.

**Mutual proximity graphs (mp):** for construction of mp graphs we first rescale the distance matrix *D* using the hubness reduction method mutual proximity (MP) (Schnitzer et al., 2012). MP rescales the original distance space so that two objects sharing similar nearest neighbours are more closely tied to each other, while two objects with dissimilar neighbourhoods are repelled from each other. MP reinterprets the distance of two objects as a mutual proximity (a probability) in terms of their distribution of distances. To compute MP, we assume that the distances $D_{x,y=1\ldots N}$ from an object *x* to all other objects in our data-set follow a certain probability distribution, thus any distance $D_{x,y}$ can be reinterpreted as the probability of *y* being the nearest neighbour of *x*, given their distance $D_{x,y}$ and the probability distribution *P*(*X*). MP is then defined as the probability that *y* is the nearest neighbour of *x* given *P*(*X*) and *x* is the nearest neighbour of *y* given *P*(*Y*):

(1) $$\begin{array}{c} MP\left(D_{x,y}\right)=P\left(X>D_{x,y}\cap Y>D_{y,x}\right). \end{array}$$

To compute MP in our experiments, we use the empirical distribution instead of assuming a functional form. Changing from similarities to distances, computation of 1-MP yields a so-called secondary distance matrix $D^{MP}$, which is then used to construct an adjacency matrix $A^{MP}$. Using $A^{MP}$, we construct a knn graph exactly as described above, which we now call a mp graph, since it is based on distances rescaled via MP. Just as the knn graph, the mp graph has an out-degree $d^{+}\left(v_{i}\right)=k$ for all vertices, and in-degrees $d^{-}\left(v_{i}\right)$ in the interval [0,N-1].

### 4.2. Graph evaluation measures

k-**occurrence (#hub, #anti, maxhub, hubness** $S^{k}$ **)**: the number of times the song occurs in the first *k* nearest neighbours of all the other songs in the database (so-called *k*-occurrence $O^{k}$) is provided as an indication of the hubness of a given song (see e.g. Aucouturier & Pachet, 2004). This *k*-occurrence is of course identical to the in-degree $d^{-}$ of a vertex. The mean of $O^{k}$ across all songs in a database is equal to *k*. Any *k*-occurrence significantly bigger than *k* therefore indicates existence of a hub. We select k=5 because our music recommender always shows the five most similar songs. As has been done before (Schnitzer et al., 2012), we define that any song with $O^{k}>5k=25$ is a hub, any song with $O^{k}=0$ is an anti-hub, any song with $O^{k}>0\;\wedge \;O^{k}\leq 5k=25$ is a so-called normal object. We compute the maximum *k*-occurrence, maxhub (i.e. the biggest hub), the number of songs of which the *k*-occurrence is more than five times *k* (#hub), and for which it is equal to zero indicating an anti-hub (#anti). To further characterise the strength of the hubness phenomenon, we use a hubness measure (Radovanović et al., 2010), which is defined as the skewness of the distribution of *k*-occurrences, $O^{k}$, of all objects in a data set:

(2) $$\begin{array}{c} S^{k}=\frac{E[{\left(O^{k}-\mu_{O^{k}}\right)}^{3}]}{\sigma_{O^{k}}^{3}} \end{array}$$

where μ and σ are the mean and standard deviation of the *k*-occurrence (in-degree) histogram, respectively, and *E* is the expectation operator. A data-set having high hubness produces few hub objects with very high *k*-occurrence and many anti-hubs with *k*-occurrence of zero. This makes the distribution of *k*-occurrences positively skewed, which we use as a measure of hubness.

**Reachability (reach)**: This is the percentage of songs from the whole database that are part of at least one of the recommendation lists, i.e. (1-(#anti/N))×100. If a song is not part of any of the recommendation lists of size k=5, it cannot be reached using our recommendation function, unless it is directly chosen as a starting song by a user.

**Number of edges (#edges)**: we give the number of edges *e* in a graph *G*, i.e. |*E*| in graph notation.

**Self-avoiding random walk ($\overset{¯}{w}$)**: to simulate user behaviour with the recommendation system, we use a so-called self-avoiding random walk approach (Madras & Slade, 1993). A random walk (see e.g. Brandes & Erlebach, 2005) ω(1,2,⋯,l) in a directed graph G=(V,E) is a Markov chain, where the probability to move from one vertex $v_{i}$ at time point *t* to vertex $v_{j}$ at time point t+1 is only conditional on the state of the system at time *t*. A self-avoiding random walk has the additional property to not allow any vertex to be member of the random walk more than once:

(3) $$\begin{array}{cc} & Pr[\omega \left(t+1\right)=v_{j}\;|\;\omega \left(t\right)=v_{i}]\\ & \;=\left\{\begin{array}{c} \frac{1}{d^{+}\left(v_{i},t\right)}\;\;\text{if}\;\left(e_{ij}\in E\right)\wedge \left(v_{j}\notin \omega \left(1\cdots t\right)\right)\\ 0\;\;\;\text{otherwise} \end{array}\right. \end{array}$$

where $d^{+}\left(v_{i},t\right)$ is the number of edges leaving vertex $v_{i}$ at time *t*. The temporal dependence of $d^{+}\left(v_{i}\right)$ arises from the self-avoiding criterion wherein $v_{i}$’s out-degree count decreases each time a node in it nearest neighbour list is visited during the random walk. This means that at every step, the random walk picks a random edge leaving the current vertex and follows it to the destination vertex of that edge, but it is forbidden to return to any vertex $v_{j}$ that already is a member of the random walk ω so far. This last condition of course renders the walk non-Markovian and is introduced to guarantee that in our simulation every song (vertex) is only visited once. After all it seems unlikely that within the course of a rather short interaction, users would return to songs they have already listened to. We simulate 100,000 self-avoiding random walks of length l=20, thereby visiting 20 songs which gives about one hour of music listening, assuming an average duration of 3  min per song. For every walk, a starting vertex is picked at random. This altogether simulates a simple interaction of users with the recommendation system, allowing us to compute average access statistics for all songs in the database. Note that $d^{+}\left(v,t\right)\in \left[0,d^{+}\left(v,0\right)\right]$ due to the self-avoiding construct, so the walk can terminate early if $v^{\prime }s$ nearest neighbours have all been previously visited.

**Strongly connected component (scc, #scc,** $\overset{¯}{scc}$ **)**: For our nearest neighbour graph, a strongly connected component (SCC) is a subgraph where every song is connected to all other songs travelling along the directed nearest neighbour connections. We use Tarjan’s algorithm (Tarjan, 1972) to find all SCC-graphs in our nearest neighbour graph with k=5. We report the size of the largest strongly connected component as a percentage of the whole database (scc), the number of additional strongly connected components (#scc) and their average size ($\overset{¯}{scc}$) in number of vertices.

ϕ-**edge ratio (** ϕ **)**: for a labelled graph (*G*, *l*), a ϕ-edge is any edge $e_{ij}$ for which $l_{i}\neq l_{j}$ (Ozaki et al., 2011), i.e. in our case for which the genres *l* of the songs corresponding to the vertices $v_{i}$ and $v_{j}$ do not match. Since our vertices can have one or two labels (genres), each ϕ-edge is assigned a value according to the percentage of non-overlapping genres between vertices:

(4) $$\begin{array}{c} \phi \left(i,j\right)=\left(1-\frac{|l_{i}\cap l_{j}|}{|l_{i}\cup l_{j}|}\right)\times 100 \end{array}$$

with $l_{i}$ ($l_{j}$) being a set of all genre labels for the song corresponding to vertex $v_{i}$ ($v_{j}$) and |.| counting the number of members in a set. Therefore, ϕ(i,j) is defined as one minus the number of shared genre labels divided by the set size of the union of sets $l_{i}$ and $l_{j}$, times 100. The latter is done to account for songs with two genre labels as compared to only one genre label. The range of values for ϕ is between 0 and 100. The ϕ-edge ratio is the average of ϕ-values for all edges in a graph. If the edges in a graph reflect the semantic meaning of its labelled vertices, it will have a low ϕ-edge ratio.

## 5. Results

Our analysis results using the evaluation indices defined in Section 4.2 are given in Table 1. We will now present the results structured into analysis of hubness, random walks, strongly connected components and semantics in Sections 5.1–5.4.

### 5.1. Hubness analysis

The knn graph, which is the basis of the FM4 Soundpark, shows considerable hubness. The number of hubs (#hub) is 291 and there are 2661 anti-hubs (#anti), which results in a very high skewness of the distribution of *k*-occurrences $S^{k}=13.97$. Hubness values of $S^{k}>1.4$ can already be seen as problematic (Schnitzer et al., 2012). As a consequence, only 65.28% of vertices are reachable. About a third of the data are therefore never being recommended. The biggest hub *maxhub* alone appears 419 times in the recommendation lists, i.e. it is recommended for 5.47% of the songs in the database. If the songs were randomly distributed across the recommendation lists, the expected number for *maxhub* would be 5 or only .065%.

Looking at the muknn graph, all hubs are gone but there is a high number of anti-hubs (4566). Hubs vanish as a consequence of the strict requirement of mutual neighbourhoods when building the muknn graph, since now every vertex can only be connected to at most five other vertices. Therefore, the number for *maxhub* is down to 5 and hubness $S^{k}$ down to 1.43. This also has the consequence that many edges from the knn graph are being deleted (#edges=5790 instead of 7665×5=38,325 for knn), resulting in the very high number of anti-hubs (4566), and a low reachability of 40.43%.

To improve this situation and promote better connectivity, we have added a minimum spanning tree to the muknn graph yielding the muknn+msp graph. Since a minimum spanning tree by definition connects all the vertices in a graph, the number of anti-hubs is zero and the reachability is at 100%. Compared to the muknn graph, the msp adds 11,826 edges yielding a total of #edges=17,616 for the muknn+msp graph. These additional edges increase the number of hubs to #hub=23. This is still much better than 291 for the knn graph but also worse than the zero hubs of the muknn graph. The biggest hub *maxhub* is also larger at 145, but still smaller than the original 419 of the knn graph. As a further consequence, hubness $S^{k}$ is back up to 10.15.

The mp graph shows a low number of 2 hubs and a moderate number of 641 anti-hubs, with a highly increased reachability of 91.62%. The biggest hub *maxhub* has a moderate size of 30 and hubness $S^{k}$ is down to only .91. Since the mp graph is essentially a knn graph built from an adjacency matrix based on rescaled secondary distances, the number of edges is identical to that of the knn graph (#edges=38,325).

**Table 1.** Analysis of knn, muknn, muknn+msp and mp graphs.

	knn	muknn	muknn+msp	mp
maxhub	419	5	145	30
#hub	291	0	23	2
#anti	2661	4566	0	641
$S^{k}$	13.97	1.43	10.15	0.91
reach	65.28%	40.43%	100.00%	91.62%
#edges	38,325	5790	17,616	38,325
$\overset{¯}{w}$	19.68	2.40	4.50	19.50
scc	29.11%	11.89%	100.00%	85.26%
#scc	408	652	0	102
$\overset{¯}{scc}$	2.87	3.36	–	2.87
ϕ	55.78%	37.79%	52.10%	52.20%

**Figure 2.**
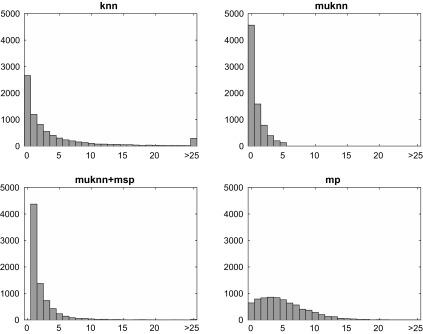
Distribution of *k*-occurrences for knn, muknn, muknn+msp and mp graphs. Notice the strong skewness in knn, muknn, and muknn+msp, while the mp graph is much more evenly dispersed with few hubs and anti-hubs.

In Figure 2, we have plotted the distribution of *k*-occurrences for the four different graph methods separately. As outlined above, a *k*-occurrence of a song is equal to the in-degree $d^{-}$ of the corresponding vertex. In the plots in Figure 2, in-degrees ranging from $d^{-}=0$ to $d^{-}>25$ are given on the x-axis, with the number of vertices exhibiting these in-degrees on the y-axis. Bars on the very left of each plot therefore show the number of anti-hubs #anti ($d^{-}=0$), bars on the far right the number of hubs #hub ($d^{-}>25$). As can be seen in the plot for the knn graph (left plot, top row), there is a considerable amount of both anti-hubs and hubs. For the muknn graph (right plot, top row), there are no in-degrees larger than 5, but at the same time also many more anti-hubs from enforcement of the mutuality criterion. Looking at the plot for the muknn+msp graph (left plot, bottom row), we can see that all anti-hubs are gone (i.e. graph has one connected component), but a few hubs have re-appeared and there exist many vertices with an in-degree equal one. These vertices with only a single edge linking to it are a direct consequence of adding a minimum spanning tree, since a single in-going edge per vertex can be sufficient to allow for full connectivity of the graph. Looking at the plot for the mp graph (right plot, bottom row), one can see a much more balanced distribution of in-degrees around the expected value of five, with much fewer anti-hubs with $d^{-}=0$ and only few vertices with in-degrees larger than 10 or 15.

### 5.2. Random walk analysis

As to the results of the random walk analysis, the average lengths of the random walks $\overset{¯}{w}$ are given in Table 1. As outlined in Section 4.2, we simulated 100,000 self-avoiding random walks with random starting vertices. Given that our database is comprised of 7665 songs, on average every vertex corresponding to a song was chosen as a starting vertex more than 10 times. The number of times every vertex was visited during all the walks ranged from 3 to 50,186 times, with both of these extreme values obtained for knn graphs. Each walk was set to a length of 20 songs, but one of the graphs (muknn) has vertices with no outgoing edges which lead to early termination of random walks. The requirement of the random walks to be self-avoiding also contributed to early termination, because it prohibits usage of edges back to a vertex that had already been used during the same walk.

As for the knn graph, the average length $\overset{¯}{w}$ is 19.68 indicating that almost all walks terminated normally, i.e. after the full intended length of 20. As outlined in Section 4.1, the out-degree $d^{+}$ of all vertices in the knn graph is equal five. A premature termination of a walk can therefore only happen if all five vertices linked via the outgoing edges have already been visited earlier in a walk, which seems to happen rather rarely. There is a quite different situation for the muknn graph, with the average walk length being only 2.40. As has been explained in Section 4.1, for muknn graphs the in- and out-degree of every vertex is equal and between zero and five. As can be seen in Figure 2 and explained above, there is a large number of anti-hubs with $d^{-}=d^{+}=0$. Such an anti-hub vertex can only be part of a random walk if it is randomly chosen as a starting vertex, which then immediately leads to termination of the walk and a length of only one. For the muknn+msp graph, the average length $\overset{¯}{w}$ is 4.50, which is some improvement compared to muknn graphs. As can be seen in Figure 2 and outlined above, muknn+msp graphs do not contain any anti-hubs but very many vertices with $d^{-}=d^{+}=1$. As an additional constraint, $d^{-}\left(v_{i}\right)=d^{+}\left(v_{i}\right)$, i.e. for every outgoing edge $e_{ij}$ from vertex $v_{i}$ there exists an in-going edge $e_{ji}$ returning to the origin vertex $v_{i}$. For the many vertices with in- and out-degree equal one this means that they become terminating vertices due to the self-avoiding property of the random walks, since a walk is prohibited to return to where it originated from and additional outgoing edges do not exist. To visualise these phenomena, imagine a linear string of nodes from any vertex. The random walk can only proceed towards the end of the string once the first step in that direction has been taken, hence it will eventually terminate. Finally for the mp graph, the average length $\overset{¯}{w}$ is 19.50, almost as high as for the knn graph. This is due to the fact the out-degree $d^{+}$ of all vertices is equal five, just as for the knn graph.

**Figure 3.**
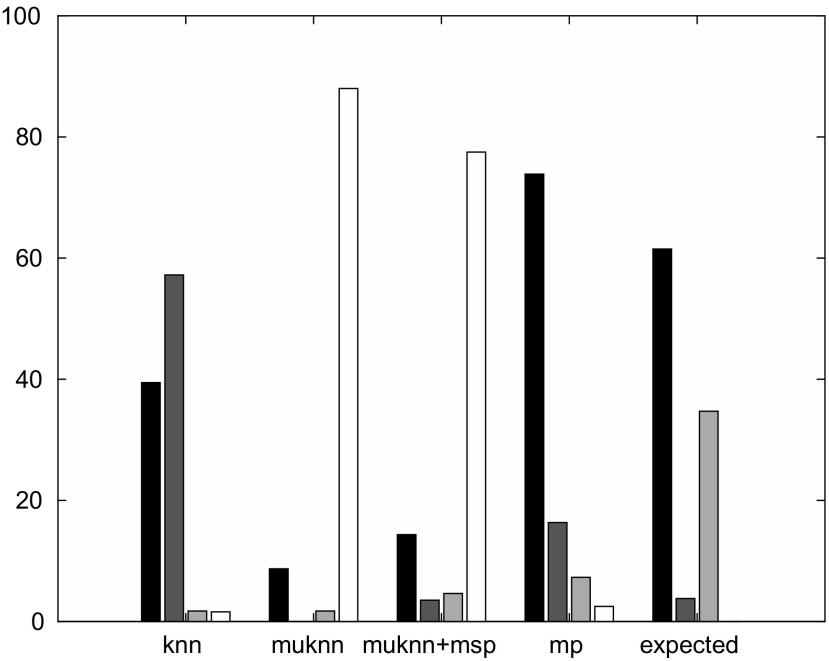
Statistics for self-avoiding random walks, black bars for normal vertices, dark grey for hubs, light grey for anti-hubs, white for missing.

In Figure 3, we have plotted the percentage of vertices visited in all 100,000 walks of length 20 which are normal, hub or anti-hub vertices (as defined at the beginning of Section 4.2) as black, dark grey and light grey bars. Since some of the walks are shorter than 20 due to premature termination (see paragraph above), we also give the percentage of overall aggregate walk length that is missing as white bars. All four bars together sum up to 100%. In addition, we show the percentage of normal, hub and anti-hub vertices in the database of songs (right-most group of bars in Figure 3, label ‘expected’). If all walks in a graph would be randomly distributed across all vertices without any preference of vertices, we would observe exactly these percentages shown as ‘expected’: 61.49% normal, 3.80% hubs, 34.72% anti-hubs.

We will now compare the results for the different graphs to these expected values. Looking at the results for the knn graph (left-most group of bars in Figure 3, label ‘knn’), we can see that more than 57% of the visited vertices are hubs, about 39% are normal vertices and less than 2% are anti-hubs. It is therefore clear that a user would spend most of their time listening to hub songs, namely 57% of the listening time would be spent listening to only 3.80% of the database (291 songs of 7665 in absolute numbers). Turning to the results for the muknn graph (label ‘muknn’), the most striking observation is that almost 88% of all walks are missing due to early termination. As for the rather small amount of actual simulated random walks, most of the time is spent at normal vertices and there are no hub vertices at all. The results for the muknn+msp graph (label ‘muknn+msp’) are only a little improved with 77% of all walks missing and most of the actual walks spent at normal vertices (14%), with hubs (4%) and anti-hubs (5%) following. The results for the mp graph shows real improvement (label ‘mp’), with less than 3% of all walk lengths missing and about 73% spent for normal vertices, 16% for hubs and 7% for anti-hubs. Comparing this to the expected values, more time should be spent for anti-hubs (34.72%) to ensure all songs can be reached, and still less for hubs (3.80%), which shows that there still is room for further improvement.

### 5.3. Strongly connected component analysis

Looking at the size of the largest strongly connected component (scc) in Table 1, one can see that it contains only 29.11% of the vertices for knn, which further decreases to 11.89% for muknn. Aside from this largest scc, there exist large numbers of additional sccs (#scc) of very small average size ($\overset{¯}{scc}$) for both knn (408 with average size of 2.87) and muknn (652 with average size of 3.36). This means that it is very likely that a user of the recommendation system will spend most of their time listening to songs within the largest scc, which comprises only one-third of the data for knn, or just about one-tenth for muknn. Due to the nature of minimum spanning trees, the largest scc of the muknn+msp graph contains all of the vertices and there are no additional smaller sccs. Therefore, every vertex in the graph can be reached from all other vertices. The scc for the mp graph is much larger than those of the knn and muknn graphs, comprised of 85.26% of the vertices, with 102 extra sccs of average size 2.87. It seems that due to hubness reduction, many vertices now connect to others instead of hubs, so the mp graph exhibits a much higher connectivity than the original knn graph.

### 5.4. Semantic analysis

Looking at the percentage of edges connecting vertices with different labels (ϕ in Table 1), we can see that all three methods, muknn, muknn+msp and mp yield improvements compared to knn. Whereas the ϕ-edge ratio is 55.75% for knn, this improves to 37.79% for muknn and slightly improves to 52.10% for muknn+msp and to 52.20% for mp. This means that the hubness reduction in all three alternative graphs also respects the semantic meaning of the data when deviating from the original knn graph. Only muknn graphs show a very strong improvement in this regard, which is due to muknn graphs having far less, but more semantically correct, edges.

## 6. Discussion

We would now like to summarise and discuss our main findings which were described in Section 5.

Our first result is that *k*-nearest neighbour graphs, as used in our music recommendation system, are negatively impacted by hubness. In corroborating earlier results (Schnitzer et al., 2012), we have found that due to hubness, only two-thirds of the music catalogue is reachable at all, while only about a third of the songs are likely to being listened to according to the size of the largest strongly connected component. Based on our random walk analysis, we found that the situation is even more severe, since almost 60% of simulated listening time is spent listening to less than 4% of the whole catalogue. All this is due to a very skewed distribution of in-degrees in the knn graph, with hub vertices having very high numbers of in-degrees.

Our second result is that mutual nearest neighbour graphs are able to reduce the negative effects of hubness, but at the cost of very poor overall connectivity. Muknn graphs do not contain any hub vertices, but very many anti-hub vertices with an in-degree equal zero. As a consequence, the reachability is down to 40% and just about 12% are likely to being listened to, which is both even lower than results for knn graphs. The random walk analysis showed that the vast majority of walks terminate prematurely, on average a simulated listening session lasts less than 3 songs instead of the intended 20. This is all due to the very strict requirement of mutuality of edges between vertices, which eliminates all hub vertices but at the same time deletes 85% of all edges from the original knn graph creating many new anti-hubs and overall poor connectivity.

Our third result is that adding a minimum spanning tree to mutual nearest neighbour graphs is not sufficient to fix the connectivity issues of muknn graphs. The resulting muknn+msp graph by its very definition guarantees full reachability of 100%, but the random walk analysis makes it clear that the enhanced connectivity still is rather weak. The large majority of walks still terminate prematurely, which on average, reduces the simulated listening session to 5 songs instead of the intended 20. The compound muknn+msp graph does contain more edges than the muknn graph, but very often what used to be anti-hub vertices are now vertices with in- and out-degree equal only one, i.e. single edges leading to and from vertices.

Our fourth result is that mutual proximity graphs, as proposed in this paper, are able to decisively reduce the negative impact of hubness on knn graphs. The mp graph shows only two small hub vertices instead of 291 (a 99% reduction), as well as only few anti-hubs resulting in a reachability of more than 90%. About 80% of songs are now likely to be listened to according to the size of the largest strongly connected component. The random walk analysis shows that only 16% of the listening time is spent on what used to be hub songs, which is a great improvement compared to the 60% as observed for knn graphs. It is important to note that mp graphs keep the exact same overall number of edges as knn graphs; therefore, the average length of simulated listening sessions is almost 20 songs, just as for knn graphs.

Our final result is that all three alternative graphs respect the semantic meaning of the data, as measured as the proportion of edges between vertices with matching genre labels. Whereas muknn+msp and mp graphs show modest enhancements in this respect, muknn graphs allow for more improvement, but at the cost of deleting so many edges that the overall connectivity is quite poor.

As to future work, the first step will be to actually replace the knn graph in the real life Soundpark music recommendation system with a mutual proximity graph. Parallel to this update of the system, we will be able to measure differences in user behaviour like an expected increase of distinct song accesses, showing the improved reachability which we already demonstrated in our off-line analysis in Section 5. This will also make it possible to measure any change of the users’ satisfaction with the updated system based on comparing questionnaires administered before and after the system update. Since mutual knn graphs can also be used for semi-supervised classification (Ozaki et al., 2011), we are also planning to employ mutual proximity graphs for this task. In the case of music recommendation, this could be useful to propagate genre labels to vertices which represent songs without genre information. It will also be interesting to explore usefulness of mutual proximity graphs in other domains where hub vertices can be found, going beyond mere MIR.

## 7. Conclusion

We have presented mutual proximity graphs, which are a new type of nearest neighbour graph able to decisively reduce hubs, vertices with extremely high numbers of edges. Whereas the related mutual *k*-nearest neighbour graphs are able to completely prevent formation of hub vertices, they result in graphs with poor overall connectivity. Adding a minimum spanning tree to the mutual knn graph to help, in this respect, only yields very little improvement. Whereas mutual knn graphs have the strict requirement of connecting only vertices which belong to each other’s nearest neighbours, mutual proximity graphs mitigate nearest neighbour asymmetries in a more flexible probabilistic way. We showed that mutual proximity graphs can improve a real-world music recommendation system, but future work should also explore usefulness of this new approach in other scenarios where hub vertices can be found.

## Footnotes

1 To be more precise, the authors analyze directed knn graphs made bi-directional through enforcing adjacency symmetry.

2 Note that the knn graph in our recommender system is a directed knn graph with an asymmetric adjacency matrix (see Section 4.1 for detail). For the remainder of the paper, knn graph refers to directed knn graphs.

3 To be more precise, updates to the database are done in a daily batch format.
